# Early Results of Using AI in Mammography Screening for Breast Cancer

**DOI:** 10.3390/jcm14217886

**Published:** 2025-11-06

**Authors:** Hadar Sandler Rahat, Tal Friehmann, Marva Dahan Shemesh, Shlomit Tamir, Eli Atar, Tzippy Shochat, Arnon Makori, Ahuva Grubstein

**Affiliations:** 1 Department of Imaging, Rabin Medical Center—Beilinson Hospital, Petach Tikva 4941492, Israel; hadarsandler@gmail.com (H.S.R.); marvada@clalit.org.il (M.D.S.); shlomitt@clalit.org.il (S.T.); elia@clalit.org.il (E.A.); ahuvag@clalit.org.il (A.G.); 2 Faculty of Medical and Health Sciences, Tel Aviv University, Tel Aviv 6997801, Israel; 3 Statistical Department, Rabin Medical Center—Beilinson Hospital, Petach Tikva 4941492, Israel; tzippysh@clalit.org.il; 4 Department of Imaging, Assuta Medical Centres, Tel Aviv 6971028, Israel; arnonma@clalit.org.il

**Keywords:** mammography, AI, breast imaging

## Abstract

**Background**: Recent advancements in Artificial Intelligence (AI) have the potential to address the challenges of mammographic screening programs by enhancing the performance of Computer-Aided Detection (CAD) systems, improving detection accuracy, and reducing false positive rates and recall rates. These systems were mostly investigated by control trials using cancer-enriched datasets and multiple readers. **Objectives**: This study aims to evaluate the real-world impact of AI integration on the performance of a breast cancer screening program. **Methods**: In January 2021, our mammography unit integrated an AI system (iCAD version 2.0) into its mammographic screening protocol. This study evaluates audit data of 31,176 mammograms interpreted between 2017 and 2021, comparing 24,373 mammograms prior to AI implementation and 6803 after the integration. Logistic regression analysis was used to assess the statistical significance of changes in key screening metrics, with a significance level of *p* < 0.05. **Results**: This study assesses the impact of artificial intelligence (AI) on mammographic screening. The cancer detection rate increased significantly from 6.2 per 1000 in 2019 to 9.3 per 1000 in 2021, with cancers detected on mammograms rising to 98%. Stage 1 cancer detection reached 100%, and the false negative rate dropped to 0%. Additionally, ductal carcinoma in situ (DCIS) detection decreased from 36.4% in 2019 to 20% in 2021. These findings highlight AI’s effectiveness in improving cancer detection accuracy and efficiency. **Conclusions**: The integration of AI into mammographic screening demonstrated promising results in improving cancer detection rates and reducing false negative rates. These findings highlight AI’s potential to enhance screening efficacy.

## 1. Introduction

Breast cancer remains the most prevalent malignancy among women globally, accounting for over 500,000 annual deaths worldwide [1]. Mammographic screening programs, widely implemented in developed countries, have demonstrated efficacy in reducing mortality rates and enhancing early cancer detection [2]. However, these programs face significant challenges, with up to 25% of mammographically visible cancers eluding detection during routine screenings [1,3,4].

They also cause a substantial workload burden for breast radiologists [2], a concern exacerbated by the general shortage of breast radiologists [5,6,7].

In response to these challenges, artificial intelligence (AI) has emerged as a promising solution. Recent advancements in AI systems have significantly improved the performance of Computer-Aided Detection (CAD) algorithms used in mammogram analysis [8]. These CAD systems are designed to identify soft tissue lesions and suspicious calcifications, potentially enhancing the accuracy and efficiency of mammographic interpretations [9].

Studies have shown that AI-assisted screening increases cancer detection accuracy [5,8,10], increases early cancer detection rates [5], decreases false positive rates and decreases recall rates [8], without increasing mammographic reading time [10]. Some studies that compared stand-alone AI-based screening to traditional radiologist screening showed AI-based screening was not inferior to radiologist screening sensitivity [11,12] and could substantially reduce the workload [11]. Furthermore, AI systems show promise in stratifying mammography exams, potentially allowing for the exclusion of radiologist review in low-risk cases [12].

However, while controlled trials and enriched datasets have provided important insights into the potential benefits of AI, there is a lack of real-world data on its implementation outside a research environment. In clinical practice, the integration of AI into mammography workflows is subject to variability in how radiologists engage with the system. Unlike in research settings—where AI outputs are always reviewed and considered—real-world scenarios may involve cases where the interpreting radiologist does not actively consult the AI findings, perceives them as time-consuming to review, or feels that low specificity undermines their diagnostic confidence. In some situations, the AI’s alerts may even contribute to an increase in BI-RADS 0 assessments due to heightened caution, potentially affecting recall rates. These factors highlight the importance of assessing AI performance not only under optimal study conditions, but also in routine, heterogeneous clinical settings where user adoption, trust, and workflow integration play crucial roles in determining its actual impact.

The purpose of this study was to evaluate real-world mammography screening performance following the implementation of an AI system, by reviewing its impact on standardized audit benchmarks (we did not prospectively collect AI individual results as would typically be conducted in a research environment). In January 2021, our breast imaging unit incorporated an AI system (iCAD version 2.0) into its reading protocol. Since then, most of the exams have been interpreted by breast radiologists with AI-assisted decision support. This article presents a preliminary report on the real-world impacts observed in our screening program’s auditing analysis following the implementation of this AI software. Our findings contribute to the growing body of evidence regarding the practical applications and outcomes of AI integration in clinical mammography settings.

## 2. Materials and Methods

**Figure 1:**
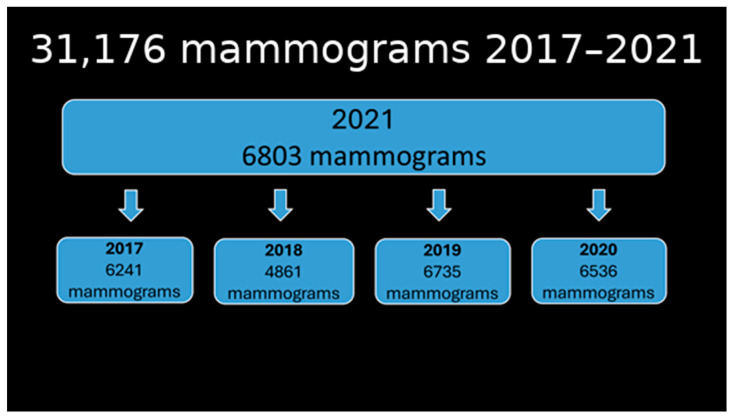
Number of mammograms in 2021 compared to previous years.

Clalit Health Services is the largest Health Maintenance Organization (HMO) in Israel. Mammography interpretations at Clalit Health Services undergo central auditing by the National Breast Program. According to the Ministry of Health policy in Israel, biennial screening mammography is offered to all women aged 50 to 74. This study is based on the audit results of mammography interpretations from the Beilinson mammography unit, covering a period of three years before and one year after implementing AI software for mammography interpretation and breast cancer risk assessment. Since the AI software was implemented in January 2021, the 2021 cohort audit was compared to previous annual mammography audits (Figure 1).

### 2.1. Software System

**Figure 2:**
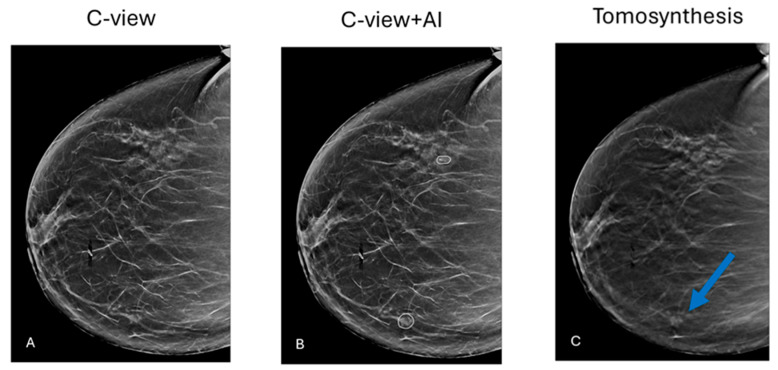
(**A**) Primary mammographic examination. There is no apparent pathology. (**B**) iCAD software detected 2 suspicious locations: a group of calcifications (lateral) and an architectural distortion (medial). (**C**) Tomosynthesis clearly shows the distortion flagged by AI. Biopsy confirmed Invasive Ductal Carcinoma.

The AI system implemented for screening is the commercially available ICAD system (iCAD; M-Vu iCAD^®^, version 3, Nashua, NH, USA). The AI system provides findings and an examination score to radiologists during assisted readings, which stratifies screenings based on breast cancer probability. It categorizes each case into four risk groups, predicting the likelihood of breast cancer diagnosis/development within the next 1–2 years based on mammography images and patient age, compared to the general population risk at the same age (Figure 2).

### 2.2. Reading Protocols

**Figure 3:**
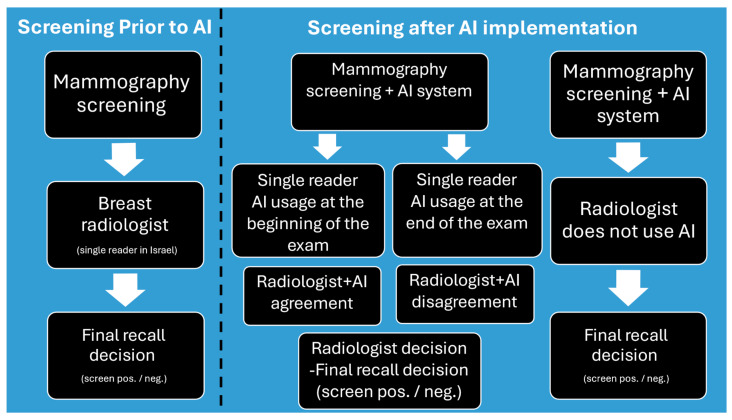
Screening protocol before and after AI implementation.

A team of fellowship-trained full-time breast radiologists, with experience ranging from 6 to 16 years, interpreted mammograms in a single-reader format. All radiologists received comprehensive training on the AI system, after which reading protocols were determined individually by each radiologist (Figure 3).

Standardized audit reports, as received from the National Breast Program’s central epidemiology unit, of Clalit Health Services included the number of screening mammograms, age distribution, true and false positives, and the cancer detection rate (CDR), and stage. These parameters were compared across the three study periods.

### 2.3. Statistical Analysis

Descriptive statistics were generated for categorical and continuous variables, and group comparisons were conducted as appropriate. Logistic regression was used to test the differences in the probability of screening between different cohorts. *p* < 0.05 was considered to indicate a statistically significant difference. SAS software version 9.4 was used for statistical analysis (SAS Institute Inc., Cary, NC, USA).

## 3. Results

### 3.1. Mammography Categories

**Figure 4:**
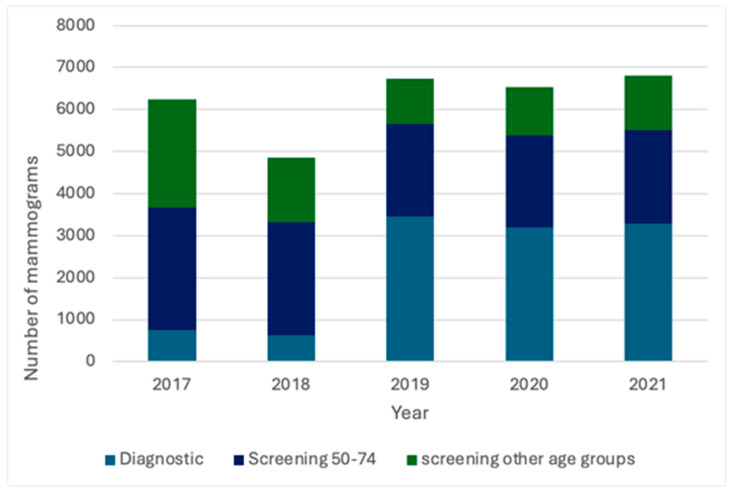
Distribution of screening and diagnostic mammograms in 2017–2021.

A total of 31,176 mammographic interpretations were included in the study, with 24,373 interpreted before and 6803 after AI implementation. Audit data from 2017 to 2021 indicated that approximately half of the mammograms in 2019–2021 were categorized as screening and half as diagnostic. In contrast, screening accounted for 87–88% of mammograms in 2017 and 2018. The number of mammograms for women aged 50–74 remained similar in 2019–2021 but was significantly higher in 2017 and 2018. The same pattern was noted for women under 50. The number of diagnostic mammograms was consistent in 2019–2021 but significantly lower in 2017–2018 (Figure 4).

### 3.2. Screening Benchmarks

The number of overall detected breast CDRs was higher in 2021 compared to 2019 and 2020 (*p* < 0.0001). CDR in the screening ages 50–74 was lower in 2021 compared to 2020 and 2019. The number of invasive malignancies found in that age range was between 4 and 7 malignancies annually. The discrepancy between all detected malignancies and those found by mammography was the lowest in 2021. The false negative rate (FNR) in 2021 was also lower than in 2019 and 2020. The number of malignancies in women aged 50–74 was similar across all years (Table 1).

### 3.3. Cancer Stage

The number of invasive cancers detected at stage 1 in screening mammograms for women aged 50–74 was highest in 2021, like 2020, and statistically significantly higher than in 2019. The proportion of ductal carcinoma in situ (DCIS) cases detected was similar in 2020 and 2021 but lower compared to 2019.

## 4. Discussion

Screening programs have long been considered effective in the early detection of breast cancer. However, the sensitivity of digital mammography (DM) is estimated to be around 87% [13]. Many studies have shown promising results for the use of AI in screening programs, with most of the reported benefits demonstrated in controlled trial environments [1,2,3,4,5,9,10,11,12,13,14,15].

Current evidence does not demonstrate an effect of AI-supported screening mammography on breast cancer survival. Existing studies are primarily diagnostic-accuracy and implementation trials, reporting process and early surrogate outcomes—such as CDR, recall and false-positive rates, positive predictive value, and workload—rather than mortality. Therefore, there is no direct evidence to date that AI use in screening mammography improves breast cancer survival. Ongoing trials with longer follow-up are required to determine its effects on interval cancer rates and mortality.

While most published studies are conducted in research settings—where every examination is known to have been reviewed by the AI system—real-world practice differs substantially. In routine clinical settings, it is often unclear whether, and to what extent, radiologists actively incorporate AI findings into their interpretations [16]. Notably, improved standardized audit benchmark results compared with readings without AI were previously demonstrated, even when the level of radiologist integration of AI outputs was unknown. Similarly, in real-world double-reading scenarios of screening mammography—including tomosynthesis—studies have likewise reported improvements in audit benchmarks [8,15].

In line with these real-world findings, our study aimed to evaluate the impact of integrating an AI system into the performance of our breast unit in routine daily use. During 2021, the first year after AI implementation, 6803 mammograms were performed, approximately half for screening and half for diagnostic purposes. Comparison of audit reports from 2017 to 2021 demonstrated a shift in the distribution between screening and diagnostic mammograms, with a progressive decrease in the proportion of screening examinations and a corresponding increase in diagnostic examinations. This change likely reflects modifications in labeling and data acquisition due to updates in the RIS (Radiology Information System), digitalization of reporting systems in both radiology and pathology, as well as personnel changes. These factors limit direct comparisons between all years; therefore, we focused on comparing 2021 with 2019–2020, which were more demographically comparable.

Several screening mammography audit benchmarks [17] improved in 2021 compared to 2019–2020. These included higher CDR, a greater proportion of cancers detected on mammography, a higher percentage of stage 1 cancers, a false-negative rate of 0% (although this exceptionally low rate may reflect registration errors), and a lower percentage of DCIS cases detected. These improvements may be attributed to the integration of the AI system, consistent with other real-world reports, where CDR was 13–18% found higher with AI support [8,15,16]. The higher-than-benchmark CDR can be attributed to the more diagnostic, rather than screening, nature of our mammography unit.

Some benchmarks were better in 2018, which may be explained by the unusually low proportion of mammograms labeled as diagnostic that year, artificially inflating the detection rate for screening examinations. The lower CDR for women aged 50–74 in 2021 may be due to the relatively small number of screening examinations in this group, limiting statistical comparisons. Notably, the COVID-19 pandemic did not result in a significant change in the overall screening numbers in our cohorts.

This study has several limitations. First, the cohort size does not meet the recommended 5000 annual screening examinations for robust audit comparisons [16]. We addressed this by comparing our performance to prior years within the same unit. Second, the final reports reflect radiologist interpretation, which may not always align with AI recommendations. Studies have shown that only 30% of radiologists regularly use AI in daily practice [17], a figure influenced by concerns over inconsistency, reduced productivity, and cost [16]. In our dataset, we could not determine the proportion of cases in which AI assistance was actively utilized, since there are no objective markers indicating whether AI was used in each case. Third, we analyzed our audits using the fixed threshold implemented by the vendor. We did not perform a detailed evaluation of the effects on NPV and recall rate across different thresholds, as has been conducted in recently published studies [15,18]. Finally, as in other real-world settings, registration, labeling, and reporting processes in radiology and pathology are not strictly standardized, which can lead to errors or inconsistencies in audit data.

## 5. Conclusions

Our study presents some of the earliest real-world results from the implementation of an AI system in our breast unit, demonstrating promising improvements in standardized screening audit benchmarks. We anticipate that these gains will persist—or potentially increase—after the adaptation period has passed. However, the clinical significance of these improvements requires further validation.

## Figures and Tables

**Table 1:** Results. Cancer detection rate (CDR), False Negative (FN), and Ductal Carcinoma In Situ (DCIS).

	2019	2020	2021
Overall cancers detected	52 *p* = 0.3	52 *p* = 0.4	64
Cancers detected on mammography	42 (80.7%) *p* = 0.05	47 (90.3%) *p* = 0.2	63 (98.4%)
Malignancy detected in screening mammography in ages 50–74	14 *p* = 0.3	8 *p* = 0.8	9
CDR	6.2/1000 *p* = 0.02	7.2/1000 *p* = 0.1	9.3/1000
CDR in ages 50–74	3.2/1000 *p* = 0.004	3.2/1000 *p* = 0.003	1.8/1000
FN in mammographic screening in ages 50–74	13% *p* = 0.02	13% *p* = 0.02	0%
Stage 1 cancers detected	57.1% *p* = 0.05	100% *p* = 0.9	100%
Percent of DCIS detected	36.4% *p* = 0.6	12.5% *p* = 0.7	20%

## Data Availability

Data is contained within the article.

## Abbreviations

Cancer detection rate (CDR), Ductal Carcinoma In Situ (DCIS), Artificial Intelligence (AI), and digital mammography (DM).
